# The binding of platinum hexahalides (Cl, Br and I) to hen egg-white lysozyme and the chemical transformation of the PtI_6_ octahedral complex to a PtI_3_ moiety bound to His15

**DOI:** 10.1107/S2053230X14014009

**Published:** 2014-08-29

**Authors:** Simon W. M. Tanley, Laurina-Victoria Starkey, Lucinda Lamplough, Surasek Kaenket, John R. Helliwell

**Affiliations:** a School of Chemistry, Faculty of Engineering and Physical Sciences, University of Manchester, Brunswick Street, Manchester M13 9PL, England

**Keywords:** platinum hexahalides, hen egg-white lysozyme, PtI_3_ ligand bound to histidine, X-ray lasers

## Abstract

This study examines the binding and chemical stability of the platinum hexahalides K_2_PtCl_6_, K_2_PtBr_6_ and K_2_PtI_6_ when soaked into pre-grown hen egg-white lysozyme (HEWL) crystals as the protein host. Direct comparison of the iodo complex with the chloro and bromo complexes shows that the iodo complex is partly chemically transformed to a square-planar PtI_3_ complex bound to the N^δ^ atom of His15, a chemical behaviour that is not exhibited by the chloro or bromo complexes. Each complex does, however, bind to HEWL in its octahedral form either at one site (PtI_6_) or at two sites (PtBr_6_ and PtCl_6_). As heavy-atom derivatives of a protein, the octahedral shape of the hexahalides could be helpful in cases of difficult-to-interpret electron-density maps as they would be recognisable ‘objects’.

## Abstract 2

The platinum hexahalides have an octahedral arrangement of six halogen atoms bound to a Pt centre, thus having an octahedral shape that could prove to be useful in interpreting poor electron-density maps. In a detailed characterization, PtI_6_ chemically transformed to a square-planar PtI_3_ complex bound to the N^δ^ atom of His15 of HEWL was also observed, which was not observed for PtBr_6_ or PtCl_6_.

## 1. Introduction   

The platinum hexahalides (K_2_PtCl_6_, K_2_PtBr_6_ and K_2_PtI_6_) each consist of an octahedral arrangement of six halogen atoms bound to a platinum centre. As heavy-atom derivatives of a protein, the octahedral shape of the platinum hexahalides make them recognisable ‘objects’ in initial electron-density map interpretations compared with single-point metal atoms which may not be recognisable in noisy difference Patterson or Fourier maps (Helliwell, 2013*b* ▶). In particular, we envisage that they could become important, for example, in X-ray laser experiments that are striving to work with ever smaller samples, currently at the microcrystal/nanocrystal size but anticipated to transition to nanoclusters (see Helliwell, 2013*a* ▶). These compounds also offer a way to solve unknown protein structures by powder diffraction involving dispersive and/or isomorphous intensity differences (Helliwell *et al.*, 2010 ▶).

K_2_PtCl_6_ and K_2_PtBr_6_ have both been studied before using soaking crystallization conditions into pre-grown hen egg-white lysozyme (HEWL) crystals; Sun and coworkers described a quick soak (∼10 min) approach and test using K_2_PtCl_6_ (Sun *et al.*, 2002 ▶), while Helliwell and coworkers undertook a time-dependent analysis of K_2_PtBr_6_ binding to lysozyme studied by protein powder and single-crystal X-ray analysis (up to 3 h soak time; Helliwell *et al.*, 2010 ▶). Both of these studies showed that these complexes bound to two sites on the protein. Site 1 is on a special position in a crevice between Arg14 in two symmetry-related molecules and site 2 is close to Ser86, Lys1 and Gln41 of one molecule in a crevice next to Pro79, Asn65 and Asn74 of a symmetry-related molecule. The platinum hexaiodide is probably of most interest as the most electron-dense complex and therefore this study scrutinizes in detail the chemical behaviour of K_2_PtI_6_ soaked into pre-grown HEWL crystals and also whether its chemical behaviour is the same as that of K_2_PtCl_6_ and K_2_PtBr_6_. The cases of K_2_PtCl_6_ and K_2_PtBr_6_ have also been studied under basically identical chemical and measurement conditions to the iodo form. An unexpected chemical behaviour of the iodo compound to form a PtI_3_ moiety bound to the His15 N^δ^ atom has been observed which was not observed with the chloro or bromo forms.

## 2. Methods   

### 2.1. Crystallization conditions   

HEWL crystals were prepared using a batch method as outlined by Blundell & Johnson (1976 ▶). 60 mg HEWL was dissolved in 1 ml 0.04 *M* acetate buffer pH 4.7 and 1 ml 10% NaCl was added to the solution. HEWL crystals were then soaked for 24 h in a 10 m*M* solution of either K_2_PtCl_6_, K_2_PtBr_6_ or K_2_PtI_6_. Each heavy-atom compound solution was obtained from a pre-made stock solution at 50 m*M* in acetate buffer.

### 2.2. X-ray diffraction data collection, protein structure solution and model refinement   

A crystal from each soaking condition was scooped into a loop using Paratone as a cryoprotectant. All X-ray diffraction (XRD) data were measured on a Bruker APEX II home-source diffractometer at an X-ray wavelength of 1.5418 Å and a fixed temperature of 100 K (Table 1 ▶). The XRD data-collection strategy used led to a high completeness of unique data, high anomalous difference completeness and a reasonable level of data redundancy. All XRD data were processed using the *PROTEUM*2 software package (Bruker AXS, Madison, WI, USA).

The crystal structures were solved using *Phaser* (McCoy *et al.*, 2007 ▶) followed by rigid-body and restrained refinement with *REFMAC*5 in *CCP*4 (Murshudov *et al.*, 2011 ▶), using the previously reported lysozyme structure with PDB code 2w1y as the molecular search model (Cianci *et al.*, 2008 ▶). The use of *Phaser* was probably not required as 2w1y is relatively isomorphous to these crystals. Model building, adjustment and refinement were carried out using *Coot* (Emsley & Cowtan, 2004 ▶) and *REFMAC*5 (Murshudov *et al.*, 2011 ▶) in *CCP*4. Ligand-binding occupancies were calculated using *SHELXL* (Sheldrick, 2008 ▶). The crystallographic and molecular model-refinement parameters for K_2_PtI_6_ are summarized in Table 1 ▶ and those for K_2_PtCl_6_ and K_2_PtBr_6_ are given in Supplementary Table S1^1^. All figures were produced using *CCP*4*mg* (McNicholas *et al.*, 2011 ▶).

## 3. Results   

### 3.1. HEWL + K_2_PtI_6_   

**Figure 1:**
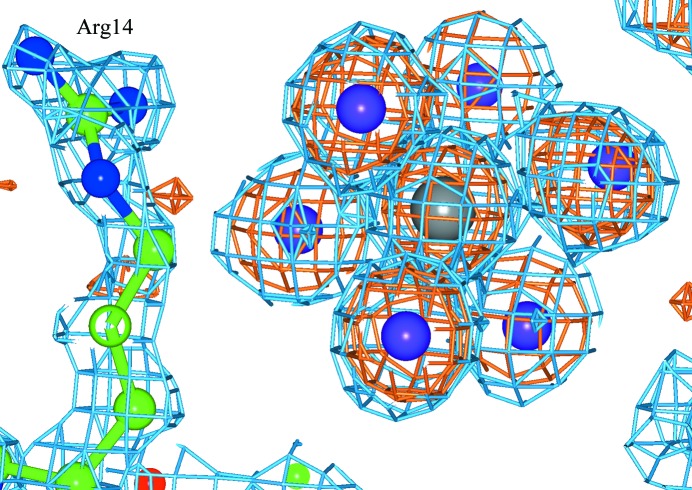
PtI_6_ binding in a special position between two Arg14 residues in symmetry-related molecules. The 2*F*_o_ − *F*_c_ electron-density map (blue) and the anomalous difference electron-density map (orange) are shown. The Pt atom is in grey and I atoms are in purple.

**Figure 2:**
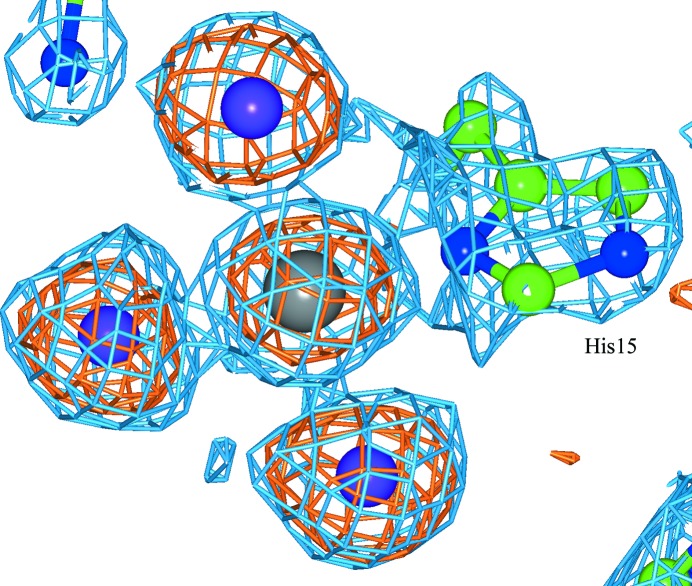
A PtI_3_ moiety bound to the N^δ^ atom of His15. The 2*F*_o_ − *F*_c_ electron-density map (blue) and the anomalous difference electron-density map (orange) are shown. The Pt atom is in grey and I atoms are in purple.

The binding of PtI_6_ to HEWL shows both similarities and differences to the previous studies involving PtCl_6_ (Sun *et al.*, 2002 ▶) and PtBr_6_ (Helliwell *et al.*, 2010 ▶). PtI_6_ binds to site 1, a crevice between two Arg14 residues in symmetry-related molecules (Fig. 1 ▶). This site is located at a special position, with a twofold axis passing through the Pt atom and two iodines. Individually refined heavy-atom occupancies, as well as whole group refined occupancies, are given in Supplementary Tables S2–S4 for each hexahalide complex. An octahedral PtI_6_ molecule is not bound in site 2, a crevice between Ser86, Lys1 and Gln41 of chain *A* next to Pro79, Asn65 and Asn74 of a symmetry-related molecule. Most interestingly, there is a new binding site that is chemically distinct comprising a PtI_3_ moiety bound to the N^δ^ atom of His15, forming a square-planar complex (Fig. 2 ▶).

### 3.2. HEWL + K_2_PtBr_6_ and HEWL + K_2_PCl_6_   

The PtBr_6_ and PtCl_6_ complexes again showed binding at two sites on the HEWL protein as seen in the previously published short soaking studies of 10 min and 3 h with HEWL (Sun *et al.*, 2002 ▶; Helliwell *et al.*, 2010 ▶). However, no binding to His15 was observed for these complexes in this or the previous studies.

## 4. Discussion   

Of most novel interest is the PtI_3_ moiety bound to His15. This is reminiscent of Zeise’s salt (PtCl_3_C_2_H_4_; Black *et al.*, 1969 ▶). This also seems to be somewhat similar, but not identical, to the results of Messori *et al.* (2013 ▶), who soaked PtI_2_(NH_3_)_2_ into pre-grown HEWL crystals for two months in DMSO medium; they described a PtI_2_(NH_3_) molecule alternating between two different binding modes.

In our companion study (Tanley & Helliwell, 2014 ▶) of cisplatin and carboplatin binding to HEWL in NaI crystallization conditions, both cisplatin and carboplatin were partially converted to transiodoplatin bound at the N^δ^ binding site of His15. This is also similar to the results that we obtained under NaBr crystallization conditions for carboplatin (Tanley *et al.*, 2014 ▶) and cisplatin (Tanley & Helliwell, 2014 ▶) and in NaCl conditions, where carboplatin was partially converted to cisplatin (Tanley *et al.*, 2013 ▶).

We have also seen a PtI_3_ *X* moiety bound to a symmetry-related molecule in the cisplatin and carboplatin NaI conditions, as shown by three anomalous difference electron-density peaks bound to the Pt centre (Tanley & Helliwell, 2014 ▶).

## 5. Conclusions   

For the octahedral PtI_6_ hexahalide molecule bound to HEWL, we see a chemical transformation to a square-planar PtI_3_ moiety bound to the N^δ^ atom of His15. This showed a different chemical behaviour to that of either the PtBr_6_ or the PtCl_6_ hexahalide complexes.

For the anticipated use with the X-ray laser, and other possible challenging-to-interpret electron-density map situations, as described in §[Sec sec1]1, PtI_6_ preserved its octahedral shape at one binding site but also appeared as a chemically transformed square-planar PtI_3_ moiety bound to a histidine N atom. This is acceptable of course when interpreting an electron-density map, but one has to know in advance that a complex of this shape is to be looked out for as well as an octahedron.

## 6. Related literature   

The following references are cited in the Supporting Information for this article: Moreno-Gordaliza *et al.* (2009 ▶, 2010 ▶).

## Supplementary Material

Supporting Information.. DOI: 10.1107/S2053230X14014009/no5054sup1.pdf

PDB reference: 4owe

PDB reference: 4owh

PDB reference: 4owc

## Figures and Tables

**Table 1:** X-ray crystallographic data and final protein model-refinement statistics for HEWL crystals soaked in K_2_PtI_6_ Values in parentheses are for the last shell.

PDB code	4owc
Data-collection temperature (K)	100
Data reduction	
Space group	*P*4_3_2_1_2
Unit-cell parameters ()	*a* = *b* = 78.69, *c* = 36.94
Crystal-to-detector distance (mm)	50
Observed reflections	135236
Unique reflections	14174
Resolution ()	27.821.62 (1.651.62)
Completeness (%)	97.8 (79.0)
*R*_merge_ (%)	0.0893 (0.1779)
*I*/(*I*)	16.2 (3.7)
Multiplicity	8.7 (2.4)
Refinement	
Cruickshank DPI ()	0.09
No. of atoms	
Protein atoms	1001
Water molecules	105
Pt and halogen atoms	14
Other bound molecules or ions†	1
Average *B* factors (^2^)	
Protein atoms	16.6
Water molecules	23.3
Pt and halogen atoms	16.8
Other bound molecules or ions†	11.5
*R* factor/*R*_free_ (%)	17.4/19.3
R.m.s.d., bonds ()/angles ()	0.02/1.81
Ramachandran values (%)	
Most favoured	96.1
Additional allowed	3.9
Disallowed	0

† The other bound atom to the protein is an Na ion.

## Footnotes

1 Supporting information has been deposited in the IUCr electronic archive (Reference: NO5054).
