# Supplementary material for: The binding of platinum hexahalides (Cl, Br and I) to hen egg-white lysozyme and the chemical transformation of the PtI6 octahedral complex to a PtI3 moiety bound to His15
Source: Acta Crystallogr F Struct Biol Commun. 2014 Aug 29;70(Pt 9):1132–4. doi: 10.1107/S2053230X14014009 (PMC4157407; doi:10.1107/S2053230X14014009)
Supplement: Supplementary file 1 [file f-70-01132-sup1.pdf]

Acta Crystallographica Section F

Volume 70 (2014)

Supporting information for article:

The binding of the platinum hexahalides (Cl, Br and I) to hen egg-white lysozyme and the chemical transformation of the PtI_6 octahedral complex to a PtI_3 moiety bound to His15

Simon W. M. Tanley, Laurina-Victoria Starkey, Lucinda Lamplough, Surasek Kaenket and John R. Helliwell

S1. HEWL+K_2PtBr_6

After soaking HEWL crystals with K_2PtBr_6 for 24hrs, an octahedral $PtBr_6$ molecule is bound to site 1 (using the naming convention of Helliwell et al 2010) which is at a special position between two Arg-14 residues in symmetry related molecules with a two-fold axis passing through the platinum atom and two bromines (Figure S1). Also, an octahedral complex is bound in site two near to Ser-86, Lys-1 and Gln-41 of chain A in a crevice next to Pro-79, Asn-65 and Asn-74 in a symmetry related molecule (Figure S2), similar to the 3hr soak study (Helliwell *et al*, 2010). However, no $PtBr_3$ moiety is bound to the Nδ atom of His-15.

A large anomalous difference electron density peak of 8σ is observed 2.4Å away from the sulphur atom of Cys-6, which is disulphide bonded to Cys-127. A platinum atom is assumed bound due to the distance from the S atom. Binding of platinum to disulphide bonded Cys residues has been previously described by Moreno-Gordaliza *et al*, (2009; 2010) using mass spectrometry to study the interaction of cisplatin with insulin.

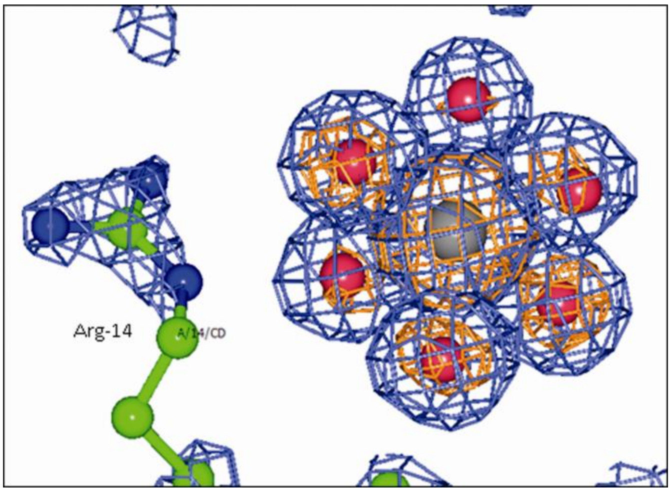

Figure S1 $PtBr_6$ binding in a special position between two Arg-14 residues in symmetry related molecules. The 2Fo-Fc electron density map (blue) and the anomalous difference electron density map (orange) are shown. The platinum atom is in grey and bromine atoms in red.

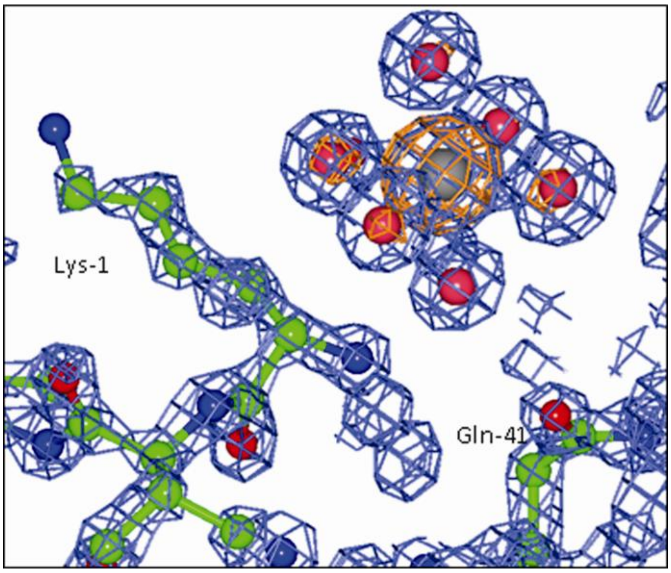

Figure S2 $PtBr_6$ molecule bound near Ser-86, Lys-1 and Gln-41 of chain A in a crevice next to Pro-79, Asn-65 and Asn-74 in a symmetry related molecule. The 2Fo-Fc electron density map (blue) and the anomalous difference electron density map (orange) are shown. The platinum atom is in grey and bromine atoms in red.

S2. HEWL+K_2PtCl_6

After soaking HEWL crystals with K_2PtCl_6 for 24hrs, an octahedral $PtCl_6$ molecule is seen bound to site 1, i.e. a special position between two Arg-14 residues in symmetry related molecules with a two-fold axis passing through the platinum atom and three chlorines (Figure S3). Also, an octahedral complex is bound in site two near Pro-79, Asn-65 and Asn-74 of chain A in a crevice next to Ser-86, Lys-1 and Gln-41 in a symmetry related molecule (Figure S2), similar to the 10min soak study (Sun *et al*, 2002). However, no $PtCl_3$ moiety is seen bound to the Nδ atom of His-15.

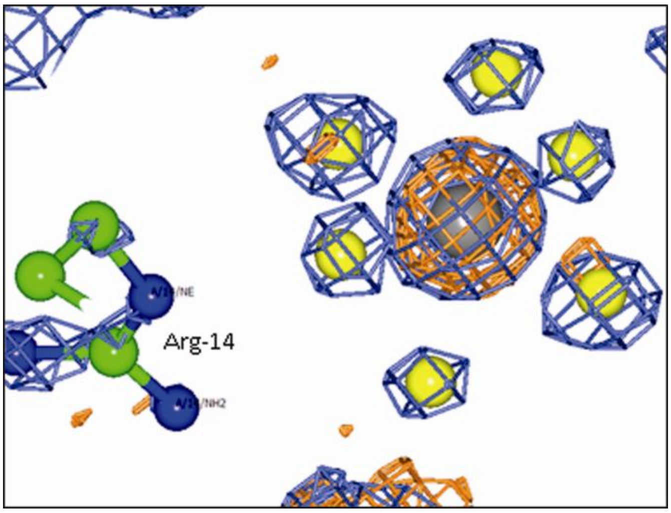

Figure S3 $PtCl_6$ binding in a special position between two Arg-14 residues in symmetry related molecules. The 2Fo-Fc electron density map (blue) and the anomalous difference electron density map (orange) are shown. The platinum atom is grey and the chlorine atoms are in yellow

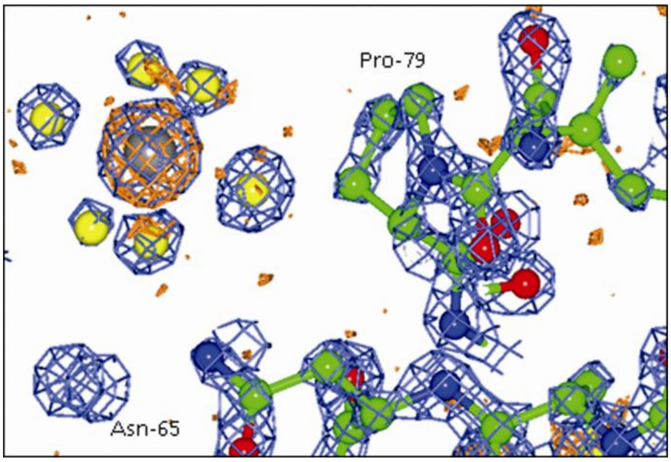

Figure S4 $PtCl_6$ molecule bound near Pro-79, Asn-65 and Asn-74 of chain A in a crevice next to Ser-86, Lys-1 and Gln-41 in a symmetry related molecule. The 2Fo-Fc electron density map (blue) and the anomalous difference electron density map (orange) are shown. The platinum atom is in grey and chlorine atoms are in yellow

Table S1 X-ray crystallographic data and final protein model refinement statistics for HEWL crystals soaked in K_2PtBr_6 and K_2PtCl_6.

	HEWL+K_2PtBr_6	HEWL+K_2PtCl_6
PDB id	4OWH	4OWE
Data collection temperature (K)	100	100
**Data reduction**		
Space group	$P4_32_12$	$P4_32_12$
Unit cell parameters (Å)	a=b= 78.66	a=b= 78.75
	c= 36.84	c= 37.16
Detector to crystal distance (mm)	50	50
Observed reflections	370257	306554
Unique reflections	17449	21867
Resolution (Å) (last shell)	55.62– 1.48 (1.52 – 1.48)	55.68-1.41 (1.48-1.41)
Completeness (%)	94.4 (30.7&)	99.8 (99.9)
Rmerge (%)	0.0585 (0.2073)	0.0941 (0.422)
<I/sigma(I)>	30.8 (1.9)	5.2 (2.6)
Multiplicity	18.7 (0.4)	5.3 (5.3)
Cruickshank DPI (Å)	0.08	0.07
Number of protein atoms	1001	1001
Average B factor (Å^2) for protein atoms	20.5	20.3
Number of water molecules	119	124
Average B factor (Å^2) for water molecules	28.7	28.1
Number of Pt and halogen atoms	23	19
Average B factor (Å^2) for Pt and halogen atoms	24.9	21.9
Number of other bound molecules or ions*	9	13
Average B factor (Å^2) for other bound molecules or ions*	34.0	37.0
**Refinement**		
R factor/ R free	18.6/22.1	18.5/22.3
RMSD bonds (Å)/ Angles (°)	0.02/1.85	0.02/2.09
**Ramachandran values (%)**		
Most favoured	96.1	94.3
Additional allowed	3.9	5.7
Disallowed	0	0

& The completeness for this dataset at a diffraction resolution of 1.52 Å is 74%.

* The other bound atoms to the protein include acetate molecules and sodium ions.

S3. Refinement of heavy atom occupancies and B factors

Refinement of heavy atom occupancies and B factors; given the different mathematical functional forms of these two parameters, provided the diffraction resolution is adequate, these two parameters can be refined simultaneously. Furthermore monitoring of how physically sensible the values obtained are form a check on the adequacy of the diffraction resolution and data quality.

Table S2 Occupancy values and anomalous difference electron density peak heights (σ) for the Pt and I atoms bound to the protein.

Atom	Nearby residues	Anom peak height (σ)	Occupancy (%)
	His-15 bound to Nδ atom		
Pt1		29.6	82
I1		20.6	66
I2		24.0	77
I3		25.7	81
	Arg-14 in a crevice between 2 symmetry related molecules		
Pt2		27.2	44
I4		20.1	41
I5		16.5	58
I6		22.0	32
I7		15.5	65
PtI_3 bound at His-15 refined as a whole molecule*			78
PtI_6 bound at Arg-14 refined as a whole molecule*			47

* The PDB files contain the refined individual occupancies as, when treated as a group, some residual electron density is observed in the Fo-Fc maps.

Table S3 Occupancy values and anomalous difference electron density peak heights (σ) for the Pt and Br atoms bound to the protein.

Atom	Nearby residues	Anom peak height (σ)	Occupancy (%)
	Arg-14 in a crevice		
Pt1		61.3	46
Br1		8.8	40
Br3		4.4	46
Br4		7.1	49
Br6		6.1	41
	Gln-41, Ser-86 and Lys-1 in a crevice		
Pt2		45.6	54
Br1N		6.3	55
Br1I		5.3	60
Br1J		4.3	38
Br1M		4.3	58
Br1K		4.0	48
Br1L		2.9	48
$PtBr_6$ bound at Arg-14 refined as a whole molecule*			47
$PtBr_6$ bound at Gln-41 refined as a whole molecule*			52

* The PDB files contain the refined individual occupancies as, when treated as a group, some residual electron density is observed in the Fo-Fc maps.

The letters next to the bromine atoms are arbitrary and denote the different atoms, similar to the different numbers.

Table S4 Occupancy values and anomalous difference electron density peak heights (σ) for the Pt and Cl atoms bound to the protein.

Atom	Nearby residues	Anom peak height (σ)	Occupancy (%)
	Asn-65, Asn-74 and Pro-79 in a crevice		
Pt1		21.3	33
Cl1		3.1	79
Cl2		3.8	39
Cl3		3.8	37
Cl4		2.6	57
Cl5		2.5	19
Cl6		3.7	55
	Arg-14 in a crevice		
Pt2		17.4	19
Cl8		0	64
Cl9		2.6	49
Cl10		3.2	42
$PtCl_6$ bound at Asn-65 refined as a whole molecule*			36
$PtCl_6$ bound at Arg-14 refined as a whole molecule*			22

* The PDB files contain the refined individual occupancies as, when treated as a group, some residual electron density is observed in the Fo-Fc maps.

S4. Other remarks on binding sites

In Helliwell *et al* (2010) a minor occupancy $PtBr_6$ site (‘site 3’) at the 20% level was seen and specifically whose occupancy did not increase with soak time up to 3 hours. In the 24hr soaking experiment reported in this new study this site 3 is not occupied at all for any of the Pt hexahalide

cases. In another case, the Cys-6-Cys-127 disulphide bond in the HEWL K_2PtBr_6 3hr study shows anomalous difference electron density, proximal to the nearest sulphur atom and therefore possibly a platinum atom. In this 24hr K_2PtBr_6 soaking experiment, there is some similar density but even less clear than the 3hr soak study. Previous mass spectrometry studies of cisplatin binding to insulin have reported cisplatin binding to Cys residues partaking in disulphide bond formation (Moreno-Gordaliza *et al*, 2009;2010). The reason for these differences between the 24hr and 3hr K_2PtBr_6 soaking studies with HEWL are not known.

Besides soaking times being different, the Helliwell *et al*, 2010 studies used glycerol as the cryoprotectant rather than paratone as reported in these current studies. However this particular difference seems unlikely to be relevant. In both the $PtCl_6$ and PtI_6 cases, no anomalous difference electron density is observed near the Cys-6-Cys-127 disulphide bond.

The occupancy values for the Pt atom in $PtBr_6$ (Table S3) at sites one and two are 46% and 54%. These have stayed at a similar occupancy as the 3hr study (Helliwell *et al*, 2010), thus increasing the soaking times from 3hr to 24hrs has not improved the binding occupancy at these two sites.

Helliwell, J.R, BellI, T.A.M, Bryant, P, Fisher, S.J, Habash, G, HelliwellI, M. Margiolaki, I, Kaenket S, Watier, Y, Wright, J, and Yalamanchilli, S. (2010) *Z. Kristallogr*. **225** 570–575

Moreno-Gordaliza, E., Canas, B., Palacios, M.A., Gomez-Gomez, M.M. (2009) *Anal. Chem* **81:** 3507-3516

Moreno-Gordaliza, E., Canas, B., Palacios, M.A., Gomez-Gomez, M.M. (2010). *Analyst* **135:** 1288-1298

Sun, P.D, Radaev, S and Kattah, M (2002) *Acta Cryst* D**58**, 1092-1098
